# Clinical Efficacy of Pulsed Radiofrequency Combined with Intravenous Lidocaine Infusion in the Treatment of Subacute Herpes Zoster Neuralgia

**DOI:** 10.1155/2022/5299753

**Published:** 2022-04-11

**Authors:** Wanyun Zhang, Chunjing He

**Affiliations:** ^1^The Department of Pain, Guihang Guiyang Hospital, Guiyang, Guizhou 550000, China; ^2^The Department of Pain, Guizhou Provincial People's Hospital, Guiyang, Guizhou 550000, China

## Abstract

**Objective:**

Under the guidance of a digital subtraction angiography (DSA) machine, via fluoroscopic imaging techniques, patients diagnosed with herpes zoster neuralgia at the subacute stage, where self-reported pain lasts between 30 and 90 days, were treated with nerve pulsed radiofrequency surgery combined with intravenous lidocaine infusion or saline infusion as control. This study explores the clinical efficacy, safety, and clinical value of the combined treatment compared with nerve pulsed radiofrequency surgery alone.

**Methods:**

In this study, 72 patients diagnosed with herpes zoster neuralgia at the subacute stage were randomly divided into two groups with matched gender, age, and clinical symptoms. Both groups received pulsed radiofrequency surgery for the affected nerve segments under DSA fluoroscopy. Five days after the operation, 0.9% saline was administered daily for five consecutive days (50 ml per day, intravenous infusion) to group A (*n* = 36), and lidocaine was administered daily for five consecutive days (3 mg per kg per day, intravenous infusion) to group B (*n* = 36). Patients with poor pain control during the treatment were given 10 mg morphine tablets for pain relief to reach visual analog scale (VAS) ≤4 points. Data of the following categories were collected: VAS score, self-rating anxiety scale (SAS) score, depression self-rating scale (SDS) score, Pittsburgh sleep quality score (PSQI), 45 body area rating scale score, skin temperature measurement using infrared thermography, analgesic drug use before and after treatment at six different time points: before surgery (*T*_0_), one day after surgery (*T*_1_), three days after surgery (*T*_2_), five days after surgery (*T*_3_), one month after surgery (*T*_4_), and two months after surgery (*T*_5_). Blood was collected from all patients in the morning before surgery and right after the last intravenous infusion of lidocaine at *T*_3_. Serum inflammatory indexes including white blood cell count, lymphocyte count, neutrophils count, erythrocyte sedimentation rate count, C-reactive protein (CRP) level, calcitonin gene-related peptide (CGRP) level, and interleukin-6(IL-6) level were determined. Lastly, the incidence of complications and adverse reactions throughout the study was recorded.

**Results:**

In total, 64 out of 72 patients completed the whole study. Two patients met the exclusion criteria in group A, one patient refused to participate, and one was lost to follow-up. Two patients met the exclusion criteria in group B, and two were lost to follow-up. Three patients in group B experienced vomiting during lidocaine treatment. The adverse symptom was relieved after symptomatic treatment. No patients in the two groups had severe complications such as hematoma at the puncture site, pneumothorax, and nerve injury. Compared with before treatment, the mean of VAS score, SAS score, SDS score, PSQI score, and skin temperature of both groups at each time point after interventional surgery were all significantly reduced. Furthermore, at each time point after surgery, the above indicators of group B patients were significantly lower than those of group A patients. After treatment, the consumption of analgesics in both groups was significantly lower than before treatment. Compared with group A, the consumption of analgesics was also significantly lower in group B. In addition, serum inflammatory indexes at the *T*_3_ time point of the two groups of patients were lower than *T*_0_. Among them, the erythrocyte sedimentation rate, CRP level, CGRP level, and interleukin-6 level of group B were significantly lower than those of group A. The incidence of postherpetic neuralgia (PHN) in group B patients (6.25%) was also lower than that in group A patients (25%).

**Conclusion:**

DSA-guided nerve pulse radiofrequency surgery combined with intravenous lidocaine infusion can effectively relieve pain in patients diagnosed with herpes zoster nerves at the subacute stage, reduce the number of analgesic drugs used in patients, reduce postherpetic neuralgia incidence rate, and improve sleep and quality of life.

## 1. Introduction

Varicella-zoster virus (VZV) infects peripheral nerves, causes spinal nerve damage in humans, and leads to a disease called herpes zoster [1, 2]. Once infected with VZV, human patients can develop herpes zoster at any time in their entire life span. The clinical manifestation of this disease is the rash of varicella form eruption in the corresponding cutaneous nerve segment area. Though the rash gradually heals, most patients still have pain in the related rash area. Herpes zoster can be defined into three stages according to the amount of time that pain lasts: acute herpes zoster (pain lasts less than 30 days), subacute herpetic neuralgia (SHN) (pain lasts between 30 and 90 days), and postherpetic neuralgia (PHN) (pain lasts more than 90 days). This type of pain is particularly evident in elderly patients with chronic diseases such as diabetes, hypertension, and immune deficiency. Most elderly patients often ignore the treatment of herpes zoster neuralgia after the lesions have been cured and consequently miss the therapeutic window. This ultimately leads to the progression toward PHN and severe impairment of psychological wellbeing and life quality.

Lidocaine is a derivative of cocaine and is a class of locally acting amide anesthetics mainly metabolized by the liver. Its primary mechanism is to block the voltage-dependent sodium-gated channels in myelinated type A and unmyelinated type C nerve fibers, thereby altering the opening and closing of ion channels and inhibiting the peripheral or central pain signal. Lidocaine has been used to treat abnormal ventricular rhythms caused by abnormal depolarization of cardiac myocyte potentials and treat chronic pain in PHN and acute pain in the perioperative period. Its metabolite, N-ethylglycine (EG), has a long-lasting analgesic effect. Systemic EG reduces the wide-dynamic-range (WDR) neuronal potentials induced by inflammatory pain and significantly improves nociceptive hyperalgesia and nociceptive abnormalities.

Pulsed radiofrequency (PRF) has been widely reported in recent years as a clinical treatment strategy for neuropathic pain. PRF treatment acts directly on the peripheral nerve or dorsal root ganglia (DRG) to improve the state of potential ectopic issuance of nerve cells and the conduction of ions in the nerve sheath membrane producing long-term inhibition of potential neuronal allocation, thus achieving long-term pain control. Compared with other interventions, PRF treatment is a less invasive, more stable, and safer procedure.

However, PRF treatment still has a high recurrence rate, limiting its clinical application. To overcome this issue, we combine RPF with continuous intravenous lidocaine infusion to evaluate the combined treatment's clinical efficacy, safety, and value.

## 2. Methods

This study was approved by the Medical Ethics Committee of Guizhou Provincial People's Hospital (Ethics No. 48), and relevant informed consent forms were signed by patients and their families before treatment [3].

**Figure 1:**
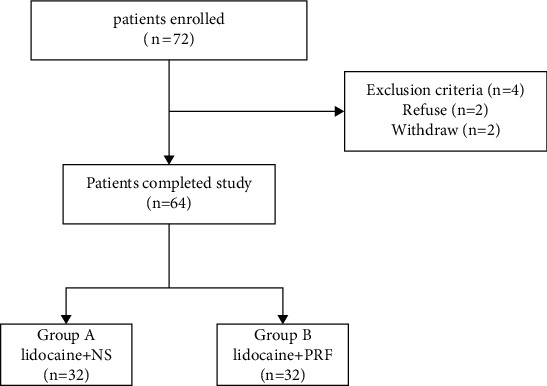
Flow diagram of randomly grouping.

Patients (50–75 years old) with subacute herpes zoster neuralgia who were hospitalized in Guizhou Provincial People's Hospital from March 2020 to December 2020 were included in this study. 72 study subjects (height 150–180 cm, weight 50–80 kg) were divided into group A (control group: 36 cases) and group B (test group: 36 cases) using the random number table method. There were 15 males and 17 females in the control group, with a mean age of 61 ± 5.25 years. In the test group, there were 18 males and 14 females with a mean age of 61 ± 4.97 years, as shown in Figure 1.

### 2.1. Inclusion Criteria

Inclusion criteria were as follows: patients who meet the diagnostic criteria for SHN and have severe burning, sharp, or electric shock-like pain, or who have nociceptive sensitivity; (2) patients with unsatisfactory pain control with medication, or patients with a VAS score ≥6 before enrollment; (3) patients aged between 50 and 75 years; (4) no severe cardiac arrhythmias; (5) no nausea, vomiting, or dizziness before enrollment; and (6) patients without serious cardiovascular, respiratory, and malignant diseases.

### 2.2. Exclusion Criteria

Exclusion criteria were as follows: (1) severe coagulation disorders; (2) cardiac arrhythmias: e.g., atrial fibrillation, atrioventricular block, and sick sinus syndrome; (3) infections at the treatment site or severe systemic infections; (4) severe spinal related diseases: e.g., scoliosis, compression fractures; (5) patients with psychiatric disorders who were unable to cooperate with treatment; and (6) patients with severe cardiovascular, respiratory and malignant diseases.

### 2.3. Surgical Methods

**Figure 2:**
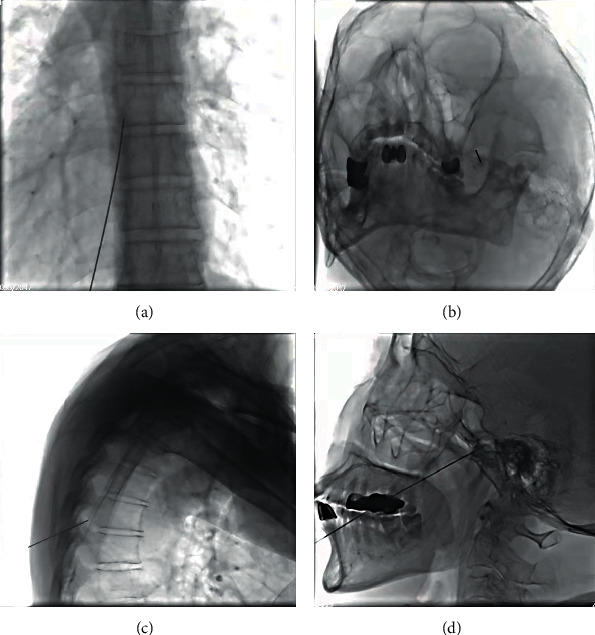
DSA guidance: DSA-guided puncture was used in each group to select a safe puncture route to avoid damage to blood vessels or lung tissue and clarify where the puncture needle would reach the target site (a, b). DSA-guided orthopantomogram of the puncture (c, d).

Patients admitted to the DSA operating room were assisted in obtaining the appropriate surgical position according to the area of herpes zoster distribution. During this procedure, the patient was monitored for changes in baseline vital signs using a cardiac monitor, and intravenous access was opened. For herpes zoster neuralgia located in the extremities or trunk, the corresponding spinal nerve segment was selected. The adjacent segment, which was the central segment, was marked and used as the target spinal nerve for radiofrequency treatment. For herpetic neuralgia of the head and face, the trigeminal nerve branches (ophthalmic, maxillary, and mandibular branches) and the glossopharyngeal nerve were selected as the target nerves for pain management according to the location of the pain area. The selected marked area was routinely disinfected and toweled, and the puncture site was anesthetized by local infiltration. When the puncture site reached the puncture anatomical landmark or target nerve, the core was removed from the RF puncture needle, and the syringe was connected for retraction. The motor stimulus (frequency 2 Hz, 1 mv) was tested to confirm successful pain replication. Pulsed radiofrequency treatment (RF parameters: 42°C, 2 Hz, 20 ms, 120 s) was applied to the corresponding nerve in the painful area. After the RF treatment was completed, the RF shock was withdrawn. The RF needle was fixed and injected with neurotrophic analgesic and inflammatory absorption promoting drugs (1% lidocaine 5 ml, compound betamethasone 1 ml, vitamin B12 diluted to 20 ml in 0.9% saline). 5 ml of the mixture was used for each spinal cord segment or peripheral nerve. After the injection, the puncture needle core was inserted again, and the RF puncture needle was withdrawn. After the procedure, the patient's vital signs were observed for 20 minutes, and after confirming that the patient's basic vital signs were stable and safe to take to the ward, the patient was monitored with ECG for 4 h, as shown in Figure 2.

### 2.4. Lidocaine Treatment

All patients were treated in a treatment room equipped with resuscitation facilities while their vital signs were monitored: heart rhythm, blood pressure, and oxygen saturation. Lidocaine was diluted to 50 ml at 3 mg/kg and infused intravenously at a rate of 25 ml/h for 2 h. After the infusion, the patient's vital signs are monitored by a doctor or nurse under cardiac supervision for 30 min and returned to the bed.

### 2.5. Morphine Consumption

During hospitalization, the patient has given 10 mg of morphine extended-release tablets orally in case of explosive pain (VAS ≥ 4, or sleeplessness at night due to pain), and an additional 10 mg of morphine extended-release tablets orally up to a maximum dose of 30 mg/d if the patient's pain is not satisfactorily controlled.

### 2.6. Follow-Up

To assess the patient's recovery, outpatient follow-up visits were conducted at 1 month and 2 months after discharge from the hospital.

### 2.7. Efficacy Evaluation

The following indicators were observed before (*T*_0_), on the first postoperative day (*T*_1_), on the third postoperative day (*T*_2_), on the fifth postoperative day (*T*_3_), on the first postoperative month (*T*_4_), and the second postoperative month (*T*_5_).

### 2.8. Primary Outcome

#### 2.8.1. VAS

A standard numerical scale used to assess the patient's subjective pain perception is widely used in clinical practice. A score of 0 < VAS < 4 is usually defined as mild pain, 4 VAS < 7 as moderate pain, and 7 VAS as severe pain.

### 2.9. Secondary Outcome

#### 2.9.1. PSQI

The PSQI scores were developed by Dr. Buysse et al. in 1989 to assess subjects' sleep quality in the last month and consist of 18 self-rated items with 7 components. In this observational study, the PSQI scores were recorded separately for both groups at each time point. The components are sleep quality, time to fall asleep, sleep duration, sleep efficiency, nocturnal sleep disturbance, hypnotic medication, and daytime dysfunction. The component scores were 0–3, and the sum of the cumulative scores was the total PSQI score, with a total score of 0–21. The higher the score, the poorer the quality of sleep.

#### 2.9.2. SAS Score

This is a 20-item scale that measures the severity of a patient's anxiety symptoms. The different items on the scale describe the patient's symptoms, and each symptom is divided into 4 levels. The total score for each item is based on the sum of the scores of the graded levels. The total score is multiplied by 1.25, and the result is rounded to the nearest whole number to give the final standard score. The higher the standard score, the higher the patient's anxiety level. According to an epidemiological survey of different anxiety scale scores in China, a standard score >70 indicates severe anxiety, 60–69 indicates moderate anxiety, 50–59 indicates mild anxiety, and a standard score >50 indicates a state of anxiety.

#### 2.9.3. SDS Score

Written by Zung et al. in the USA in 1965, this scale evaluates patients' depressive symptoms in terms of different aspects such as mental disorders, psychoaffective disorders, movement disorders, and somatic disorders. The scale is assessed and scored in much the same way as the SAS, with a standard score >70 indicating major depression, 63–72 indicating moderate depression, and 53–62 indicating mild depression, according to the scale score.

#### 2.9.4. IRTI

**Figure 3:**
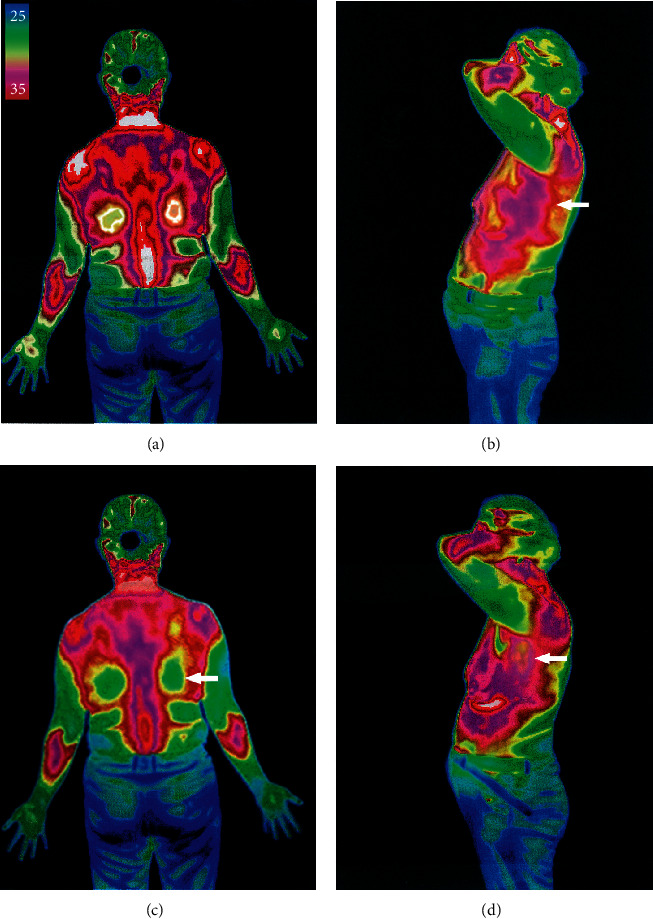
Comparison of infrared thermograms at *T*_2_ and *T*_0_ for typical cases in the group treated. (a) Body surface skin temperature on the back of the chest at *T*_0_, 35.5. (b) Body surface skin temperature in the right axilla at *T*_0_; the arrow indicates the pretreatment infrared thermogram of the painful area, showing a high body surface temperature signal at 35.6°C. (c) Body surface skin temperature on the back of the chest at T2, 34.5. (d) Body surface skin temperature in the right axilla at *T*_2_. The arrowed area shows a relatively low-temperature signal of 34.3°C on infrared thermography.

The main principle of infrared thermography is to use thermal imaging technology to test the temperature of the subject's body surface and display the temperature of each body part in different colors so that the observer can intuitively understand the temperature distribution of the subject's body surface [4]. Its simplicity and noninnovative functional imaging technology advantage can quickly reflect the subject's local microcirculatory status [5], inflammatory response, and other abnormal physiological changes. The scanning and measurement areas are usually positioned after 15 minutes of resting at a room temperature of 23–24°C, as shown in Figure 3 [6].

#### 2.9.5. 45 Body Area Rating Scale

The body area rating scale divides the body surface area into 45 areas, each marked with an area number, including 22 anterior areas and 23 posterior areas. Patients are usually asked to mark their pain area in the corresponding area, which is scored as 1 and the rest as 0. The overall pain area score is added to the patient's total pain area.

#### 2.9.6. Serum Inflammatory Index

The blood samples were collected early in the morning at *T*_0_ and after the intravenous infusion of lidocaine at *T*_3_ in both groups. Blood samples were collected to determine sedimentation, leukocytes, lymphocytes, neutrophil assay, CRP, IL-6, and CGRP at both time points.

### 2.10. Statistical Methods

GraphPad Prism 8.3 (Ver 8.3, GraphPad Software, San Diego, USA) statistical software was used for data analysis. Statistical data were analyzed using the *χ*^2^ test, and data that met the normal distribution were expressed as mean ± standard deviation ($\bar{x}$ ± SD). For data that met the normal distribution and chi-square, within-group and between-group differences were compared using repeated measures two-factor ANOVA. Nonnormally distributed measurements were tested using the rank-sum test. Differences between two groups at the same time point were compared using multiple comparisons, corrected for *α* values using the Bonferroni correction. Two independent sample *t*-tests were used to compare serum inflammatory parameters before and after treatment in the two groups. *P* < 0.05 indicates that the difference is statistically significant.

## 3. Presurgery Patient's Characteristics

There was no statistically significant difference in the general information of the two groups of patients before interventional surgery, such as gender, age, course of the disease, and affected parts, *P* > 0.05 (Table 1).

## 4. VAS Scores before and after Surgery

**Figure 4:**
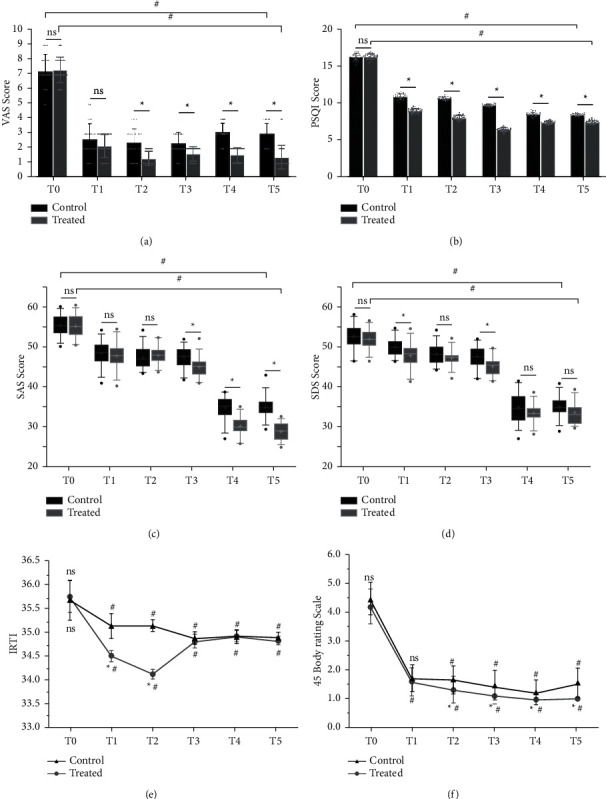
(a–f) Comparison of clinic scores at different time point of before and after PRF in the two groups. Control (PRF + NS) and treated (PRF + lidocaine); PRF = pulsed radiofrequency; NS = normal saline. Results are presented as mean ± SD. Compared to group control at a simultaneous point, ^*∗*^*P* < 0.05. The treatment group was compared to before surgery, ^#^*P* < 0.05.

There was no statistical difference (*P* > 0.05) in the pain scores between the two groups before treatment (*T*_0_). (1) A statistically significant comparison of VAS scores between the two groups of patients (*F* = 111.5, *P* < 0.05). (2) The VAS scores at *T*_0_ and *T*_1_ were not statistically significant (*P* > 0.05). Still, the VAS scores at the rest of the time points were statistically significant (*P* < 0.05), and the VAS scores of patients in group B decreased more significantly. The trend analysis of VAS scores over time was statistically significant in both groups (*F* = 1.553, *P* < 0.05), as shown in Table 2 and Figure 4(a).

## 5. PSQI Scores before and after Surgery

There was no statistically significant difference in PSQI scores between the two groups before surgery (*T*_0_) (*P* > 0.05). Comparing the PSQI scores of patients at the rest time points of postoperative treatment, the following were obtained: (1) the difference in PSQI scores between the two groups was statistically significant (*F* = 8039, *P* < 0.05); (2) the difference in PSQI scores evaluation between the two patient groups was statistically significant (*F* = 3430, *P* < 0.05); (3) the comparison of PSQI scores between two groups at the same time point was statistically significant (*P* < 0.05); and (4) the difference in the trend of change of PSQI scores between the two groups at different time points was not statistically significant (*F* = 0.929, *P* > 0.05), as shown in Table 3 and Figure 4(b).

## 6. SAS and SDS Score before and after Surgery

There was no statistically significant difference between the SAS scores of the two groups at *T*_0_*P* > 0.05) and no statistically significant difference between *T*_1_ and *T*_2_ after treatment compared to *T*_0_, and the SAS scores of both groups were lower than *T*_0_ at *T*_3_, which was statistically significant (*P* < 0.05), and the SAS scores of group B were lower than those of group A. Comparing the SAS scores of patients at different time points, we found that (1) the SAS scores of the two groups at different time points were statistically the difference was statistically significant (*F* = 841.7, *P* < 0.05). (2) The difference between the SAS scores of the two groups of patients was significant (*F* = 66.59, *P* < 0.05). (3) Comparing the SAS scores of the two groups of patients at the same time points, there was no significant difference between the *T*_0_, *T*_1_, and *T*_2_ time points (*P* > 0.05), while the comparison of SAS scores at the remaining time points was statistically significant (*P* < 0.05). (4) Comparing the temporal trend of SAS between the two groups, there was no statistical difference (*F* = 1.071, *P* > 0.05), as shown in Table 3 and Figure 4(c).

There was no significant difference in SDS scores between the two groups (*P* > 0.05). SDS scores at other time points were compared as follows: (1) Comparison of SDS score index score between the two groups at different time points (*F* = 593.6, *P* < 0.05), and comparison of SDS score between the two groups (*F* = 38.82, *P* < 0.05), the difference was statistically significant. (2) SDS score of the two groups at the same time point was statistically significant only at *T*_1_ and *T*_3_ (*P* < 0.05). (3) There was no statistical difference in SDS score between the two groups over time (*F* = 0.9016, *P* > 0.05), as shown in Table 3 and Figure 4(d).

## 7. IRTI before and after Surgery

There was no statistically significant difference in pain area temperature between the two groups of patients before treatment (*P* > 0.05). There was a statistically significant difference in pain area temperature between the two groups of patients after treatment. There was a statistically significant difference between the two groups (*F* = 235.9, *P* < 0.05 and *F* = 154.6, *P* < 0.05in body surface temperature within and between the two groups, and the decrease in body surface temperature in the pain area during lidocaine infusion in the experimental group were more significant than that in the control group, as shown in Table 3 and Figure 4(e).

## 8. 45 Body Rating Scale before and after Surgery

There was no statistically significant difference in pain area rating between the two groups before surgery (*T*_0_) (*P* > 0.05), and there was a statistically significant difference in pain area rating between the two groups (*F* = 492.8, *P* < 0.05) and between the groups (*F* = 46.1, *P* < 0.05). The pain area score at the T_2_–T_4_ time point decreased with time. The pain area score decreased more significantly in the test group than in the control group. The difference was statistically significant (*P* < 0.05), as shown in Table 3 and Figure 4(f).

## 9. Morphine Consumption before and after Surgery

**Figure 5:**
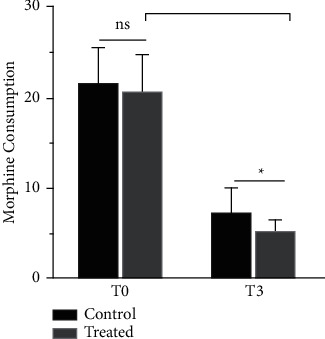
Comparison of morphine consumption in the two groups, ^*∗*^*P* < 0.05. Compared with before surgery, ^#^*P* < 0.05.

Morphine consumption during hospitalization was not statistically different between the two groups before treatment(*T*_0_) (*P* > 0.05). Postoperative morphine consumption was statistically different between the two groups (*P* < 0.05). There was a statistical difference in morphine consumption within (*F* = 703.3, *P* < 0.05) and between (*F* = 7.441, *P* < 0.05) the two groups of patients (Table. 4 and Figure 5).

## 10. Serum Inflammatory before and after Surgery

**Figure 6:**
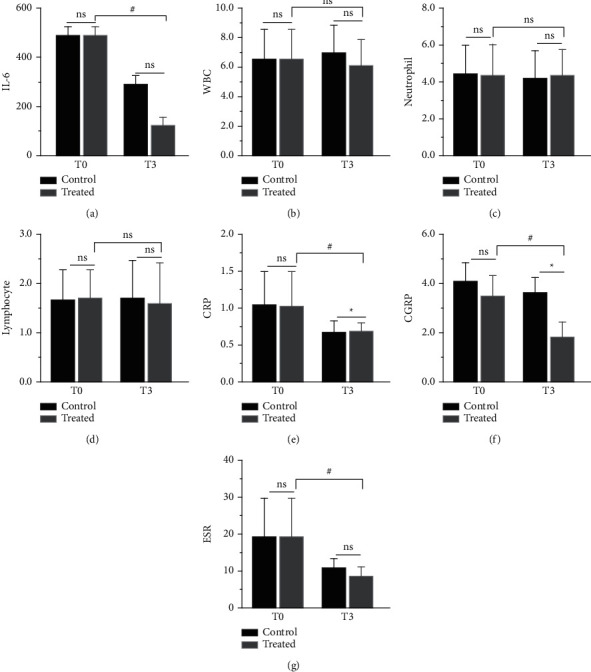
(a–g) Comparison of serum inflammatory indexes at T0 and T3 time points of before and after PRF in the two groups. Control (PRF + NS) and treated (PRF + Lidocaine); PRF = pulsed radiofrequency; NS = normal saline. Results are presented as mean ± SD. Compared to group control at a simultaneous point, ^*∗*^*P* < 0.05. The treatment group was compared to before surgery, *P* < 0.05.

There was no statistically significant difference in the (*T*_0_) serum inflammatory indexes between the two groups of patients before treatment (*P* > 0.05). There was no statistically significant difference in the white blood cell count, lymphocyte count, and neutrophil count between the two groups after treatment (*T*_5_) (*P* > 0.05). There was a statistically significant difference in serum inflammatory indexes such as hypersensitivity reactive protein, sedimentation, calcitonin, and interleukin-6 between the two groups after treatment (*T*_5_) (*P* < 0.05), as shown in Table 5 and Figures 6(a)–6(g)).

## 11. PHN Incidence Rate before and after Surgery

**Figure 7:**
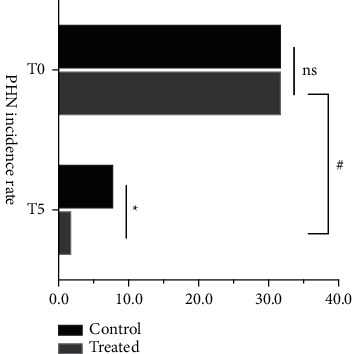
Comparison of the incidence of post-herpetic neuralgia in the two groups, ^*∗*^*P* < 0.05. Compared with before surgery, ^#^*P* < 0.05.

There was a statistical difference in the incidence rate of PHN before and after surgery between the two groups (*P* < 0.05), with 8 patients in group A and 2 patients in group B. The incidence rate of PHN after surgery was 25% and 6.25% in groups A and B, respectively. Of these, patients with a VAS score of 4 accounted for 16.25% of the overall ratio (Table 6 and Figure 7).

## 12. Adverse Events

During the treatment, one patient in the control group experienced dizziness during lidocaine infusion, and three patients in the treated group experienced vomiting during lidocaine infusion, which was relieved after symptomatic treatment; no severe complications such as lidocaine poisoning, puncture site infection, hematoma, pneumothorax, and nerve injury occurred during the treatment.

## 13. Discussion

PHN is a common clinical neuropathic pain disorder [7], usually caused by varicella-zoster virus infection when the body is immunocompromised. The HZ susceptible population is often older people between 50 and 70 years of age, with an incidence of about 3–5‰ [8]. With the progress of the aging of the society's population, epidemiological surveys show that the lifetime risk of developing HZ is about 20–30%, and the incidence is more significant in women (6.3%) than in men (5.1%), so the incidence of herpes zoster neuralgia and hospitalization rates show a yearly increase [9]. Some patients may suffer from anxiety, depression, and other emotional disorders due to pain, which seriously affects patients' physical health, psychological health, and quality of life. Patients with clinically compatible symptoms, older than 50 years of age and with a duration of more than 3 months are usually used as diagnostic criteria for clinical PHN. However, some scholars still define herpes zoster of less than 1 month as acute herpes zoster and PHN of 1–3 months duration as subacute herpes zoster [10].

Most patients are often cured of skin damage after regular antiviral or immunotherapy. Still, some patients have a prolonged course of disease due to untimely treatment of early herpes zoster or moderate to severe neuropathic pain that may persist for months or years after the herpetic lesions have healed [11]. Most patients live with severe pain for years and years, seriously affecting their sleep quality and daily life. Patients with severe pain may have negative mood changes, such as anxiety, depression, and other severe psychological disorders, burden society, and families [12]. With age, changes in the living environment, and a decrease in immunity, the latent nerve virus in the dorsal roots of the spinal cord can be reactivated, causing inflammation, hemorrhage, and necrosis of the nerve roots, which can lead to ectopic potentials, abnormal opening of nerve ion gating channels, and in cases of prolonged disease, even central sensitization, causing intractable and severe pain [13]. Patients during this period often have a combination of moderate to severe pain in the nerve distribution area. This type of pain, if not well-controlled, is often an important risk factor for the development of post-herpetic neuralgia. In recent years, some studies have shown that some patients may progress to PHN even after early treatment with medication, making the timing and choice of treatment for herpetic neuralgia particularly important [14].

The clinical treatment of herpetic neuralgia is complex. In the treatment of neuropathic pain, the pain is often poorly controlled, with individual differences in outcome, and minimally invasive interventions combined with pharmacological treatment are often used to achieve better results [13, 15, 16]. Hermanns et al. [17] have shown that lidocaine is a commonly used sodium channel blocker. Its mechanism of action is mainly as follows: (i) blocking sodium channels in nerve cells, reducing the inward flow of extracellular sodium, weakening the voltage-dependent sodium-gated channels in myelinated class A and unmyelinated class C nerve fibers, thus changing the open and closed state of ion channels. This alters the opening and closing of ion channels and inhibits the transmission of peripheral or central pain signals [18]. (ii) Lidocaine has been used to treat abnormal ventricular rhythms caused by abnormal depolarization of cardiomyocyte potentials and the treatment of chronic pain in PHN and acute pain in the perioperative period [19]. (iii) Its metabolite, N-ethylglycine (EG), has a longer-lasting analgesic effect, while systemic EG reduces the wide-dynamic-range (WDR) of the spinal dorsal horn induced by inflammatory pain [20]. The systemic EG can reduce the Wide-dynamic-range (WDR) neuronal potentials induced by inflammatory pain and significantly improve nociceptive hyperalgesia and nociceptive abnormalities.

Due to the hypoxia of nerve root fibers, inflammatory material deposition and viral invasion, can lead to nerve cell membrane potential changes and nerve fiber damage and then induce the ectopic issuance of potential and the oscillatory potential phenomenon of neurons; all these factors are involved in the formation mechanism of neuropathic pain and central sensitization [21]. After injury stimulation, peripheral nerve injury receptors receive signals, and voltage-dependent Ca^2+^ receptors on the surface of DRG cells are activated [22], and Ca^2+^ ion channels are activated to transmit signals to the dorsal horn of the spinal cord via axons, which integrate pain signals and upload them to the center, an essential mechanism for central sensitization and chronic pain development [23]. In the case of minimally invasive interventions, pulsed radiofrequency treatment can act directly on the peripheral nerve or DRG. DRG-pulsed radiofrequency treatment can improve the state of the issuance of nerve cells and the conduction of ions in the nerve sheath membrane, producing long-term inhibition of neuronal potential issuance, thus achieving long-term pain control [24]. Pulsed radiofrequency treatment is one of the much less invasive, more stable, and safer than any other surgical procedure, thus making it one of the common treatments used by clinicians [25].

Our study observed a decrease in pain scores in both groups compared to the pretreatment period. The trend graphs show more clearly that the VAS score, PSQI score, SDS score, and SAS score all tended to decrease significantly with time after treatment. The VAS score and PSQI score reduced most significantly at the postoperative period (*T*_3_), indicating that the treatment was effective in both groups and that the effect was quicker and resulted in better pain control; compared with the control group, the VAS score, PSQI score, SAS score, and SDS score decreased more significantly at different time points in the test group than in the control group (Table 3 and Figure 4). The VAS scores of patients in Group B were lower than those of Group A at different points in time, suggesting that continuous postoperative lidocaine infusion was more effective and sustained the reduction in pain levels. In line with the decreasing trend in VAS scores, the PSQI scores of patients in group B were lower than those of group A at different points in the postoperative period, suggesting that postoperative lidocaine treatment was effective in improving sleep quality and that the improvement in sleep quality lasted longer than in the control group, which is consistent with the results of the trial by Huang et al. [26]. This may be due to improved neuropathic pain or central sensitization in patients and the fact that continuous infusion of lidocaine effectively raised patients' pain thresholds and reduced their subjective pain perception, thus improving more effective control of outbreak pain and improving the quality of patients' sleep. For the improvement in patient behavior, the SAS and SDS scores of patients in group B did not improve significantly at 1 and 3 days postoperatively compared to group A. Although the patients' short-term pain was controlled, this did not improve the psychological state of patients suffering from long-term pain very well. The SAS scores at 1 and 2 months postoperatively were significantly better than those of group A, indicating that the effective relief of long-term pain through the continuous postoperative infusion of lidocaine was beneficial in improving the patients' anxiety and depression in the long term. Interestingly, some scholars have found [27] that in patients who have been diagnosed with PHN, especially those with a long course of PHN, a single treatment modality for pain does not improve the patient's anxiety and depression status well and often requires a combination of treatment with antianxiety and antidepressant drugs, contrary to the findings of this trial. Therefore, early intervention of treatment modalities better confirms the extreme importance of the timing of treatment for herpes zoster neuralgia and confirms that early treatment improves patients' dependence on psychotropic drug use.

PHN is a distinct category of neuropathic pain. It has been confirmed in several previous studies [28] that the chance of PHN is often closely related to the treatment of early pain and the recovery of autoimmunity. When the body's immune function is abnormal, the virus latent in the DRG or peripheral nerves (PN) can be reactivated, triggering herpes zoster [12, 29, 30]. It has been confirmed that the abnormal immune function of the body during this period, the suppression of specific cellular immune function, the inflammatory response of immune cells, the decreased regulation of immune response mechanisms, and the viral invasion of nerves leading to a local inflammatory response in the nerve cell sheath and abnormal [31], persistent signal afferents from spinal neurons are important pathophysiological mechanisms that induce herpes zoster neuralgia. Among others, Facchini. et al. demonstrated [16] that peripheral blood proinflammatory plasma factors are usually altered in patients with early herpes zoster and that the changes correlate with the severity of inflammation. For example, serum levels of hematocrit, calcitonin, CRP, and IL-6 are closely associated with the incidence of PHN and the formation of central sensitization. However, the correlation between the levels of proplasma inflammatory factors and PHN incidence in the treatment of subacute herpes zoster is still underresearched [32].

Our study observed that before treatment (*T*_0_), patients in both SHN groups had higher than normal levels of serum inflammatory indexes. In patients treated by lidocaine intravenous infusion combined with pulsed radiofrequency (*T*_5_), among the serum inflammatory index manifestations, inflammatory factors such as sedimentation, calcitonin, CRP, CGRP, and IL-6 decreased with remission, and to a more significant extent than in group A(Table 5 and Figure 6). Consistent with the literature, early pain control during the treatment of subacute herpes zoster improved the level of serum inflammation in patients [33]. When comparing patients' opioid pain medication (Table 4 and Figure 5) consumption at the same time point as the serum inflammatory index, morphine extended-release tablet consumption was significantly lower in group B than in group Ke et al. [34] confirmed that pulsed radiofrequency treatment significantly reduced pain medication use by evaluating pulsed radiofrequency with the evaluation of pain medication use. The present study further demonstrated that continuous postoperative intravenous infusion of lidocaine resulted in more significant pain relief than the single postoperative pulsed radiofrequency treatment groupo. Therefore, it was more effective in reducing patients' pain medication use and their dependence and addiction to such medication. The trend graph shows that as the VAS scores decreased in both groups, the PSQI scores decreased more significantly in the early stages of pain control, and the quality of sleep improved as a result of pain control. There was also a corresponding decrease in the patient's local pain area temperature and the patient's pain area, during treatment, in patients who received postoperative intravenous lidocaine, there was also a corresponding decrease in regional local skin temperature [35] (measured by thermal imaging) and pain area (measured by 45 body area rating scale) [36] in the control group, which may be associated with improved neuroinflammation in patients. Early pain control may have lasting effects on pain control. The patient's distant SAS and SDS scores were also reduced compared to the pretreatment period. Thus, effective pain control can improve sleep quality and the patient's psychological state, regulate immune function and restore damaged nerve function [18, 37].

This study treated subacute herpes zoster neuralgia by pulsed radiofrequency combined with lidocaine infusion. We compared the efficacy of the combined treatment with the correlation of serum inflammatory indexes to see whether the combined treatment modality could effectively reduce PHN incidence. In this study, 64 cases of subacute herpes zoster neuralgia were included, 31 women and 33 men. There were 12 cases in the neck, 22 cases in the chest and back, 23 cases in the lumbar region, and 7 cases in other areas, with no statistical difference in general information. Patients in the group were treated with radiofrequency nerve pulses in the pain-inflicted corresponding regions. The patients' pain was recorded and scored using the VAS before admission, and according to the scores, most patients had moderate-severe pain. Preoperative morphine hydrochloride extended-release tablets were used for pain control. Some prevalence studies have reported [38] that the prevalence of herpes zoster neuralgia is higher in women than in men, which is not consistent with the results of this study, which may be related to the small sample size selected for this trial to be verified by increasing the sample size subsequently.

Our study of patients who followed up at 2 months postoperatively found that the VAS score at the 2-month postoperative time point was the follow-up endpoint. The number of patients with pain with a VAS >3 in group A and group B were 8 and 2, respectively, representing 25.0% and 6.25% of each group(Table 6 and Figure 7). This represents 15.62% of the overall sample. This shows that the overall PHN incidence is lower than the PHN incidence in the epidemiological survey, further confirming the need for aggressive treatment for patients with SHN. The results also showed that PHN incidence was significantly lower in group B than in group A. Therefore, this trial also confirmed that in patients with SHN, pulsed radiofrequency combined with lidocaine treatment significantly reduces PHN incidence and improves the prognosis of patients. Based on the results of the trial, we speculate that this may be due to the short history of the patients, the fact that most patients do not have severe neuropathic damage, the fact that central sensitization mechanisms have not yet developed, and the fact that patients treated by intravenous infusion of lidocaine have reduced perineural inflammation, which reduces the release of pain mediators and thus effectively reduces the incidence of chronic pain. Therefore, the use of pulsed radiofrequency in combination with lidocaine in subacute herpes zoster neuralgia is of great value.

In conclusion, the combination of pulsed radiofrequency and lidocaine was effective in treating subacute herpes zoster neuralgia not only in treating the patient's pain but also in reducing the patient's use of analgesic medication [39] and in restoring normal serum inflammatory factor levels and improving the patient's psychological state of anxiety and depression. There are still shortcomings in this study, mainly due to the small sample size and the need to continue to complete data collection, and the short follow-up period of only 2 months, which lacks a long follow-up period. Therefore, further evaluation of the efficacy needs to be conducted at a later stage with multiple centers and an increased sample size to provide a more concrete answer [40].

## Figures and Tables

**Table 1:** Comparison of general information between two groups of patients.

Parameters	Control	Treated	*t*-value	*P* value
Age	63.70 ± 2.94	66.05 ± 2.08	83.53	0.74
Sex (male/female)	32	32	0.5630	0.45
Male	18 (54.55%)	15 (45.45%)		
Female	14 (45.16%)	17 (54.84%)		
Duration (days)	50.10 ± 9.86	51.46 ± 8.46	40.67	0.37
Involved nerve				
Cervical nerve (%)	9 (28.1%)	3 (9.4%)		
Thoracic nerve (%)	11 (34.4%)	11 (34.4%)		
Lumbosacral nerve (%)	8 (25.0%)	15 (46.9%)		
Others (%)	4 (12.5%)	3 (9.4%)		
Comorbidities	14	16		0.46
Diabetes (%)	6	8		
Hypertension (%)	6	5		
Immune disease (%)	2	3		

*Note.* There is no statistically significant comparison between the general data of the two groups (*P* > 0.05).

**Table 2:** Comparison of VAS scores between two groups of patients at different times.

	*T*_0_	*T*_1_	*T*_2_	*T*_3_	*T*_4_	*T*_5_
Control	7.21 ± 1.15	2.47 ± 0.75^a^	3.25 ± 0.46^a^	2.37 ± 0.75^a^	3.12 ± 0.61^a^	3.03 ± 0.82^a^
Treated	7.46 ± 1.07	2.12 ± 0.15^a^	2.85 ± 0.14^ab^	1.65 ± 0.48^ab^	1.59 ± 0.498^ab^	1.43 ± 0.504^ab^
*t* value	0.635	2.223	5.240	3.652	7.780	8.980
*P* value	>0.05	<0.001	0.092	0.0002	<0.0001	<0.0001

*Note.* Comparison with *T*_0_, ^a^*P* < 0.05; comparison with simultaneous point A group^b^*P* < 0.05.

**Table 3:** Secondary outcome between the two groups of patients before and after surgery.

	PSQI		SAS		SDS		Tsba		IRTI	
	Control	Treated	Control	Treated	Control	Treated	Control	Treated	Control	Treated
*T*_0_	16.35 ± 0.43	16.36 ± 0.37	55.39 ± 2.83	55.16 ± 2.31	52.42 ± 2.83	51.60 ± 2.31	4.46 ± 0.56	4.18 ± 0.59	35.68 ± 0.26	35.76 ± 0.33
*T*_1_	10.98 ± 0.36^a^	9.08 ± 0.30^ab^	48.35 ± 2.17^a^	47.71 ± 3.12^ab^	49.73 ± 2.17^a^	47.57 ± 3.12^ab^	1.71 ± 0.45^a^	1.59 ± 0.49^a^	35.14 ± 0.12^a^	34.50 ± 0.11 ^ab^
*T*_2_	10.74 ± 0.20^a^	8.17 ± 0.30^ab^	47.31 ± 2.37^a^	47.94 ± 1.82^a^	48.01 ± 2.37^a^	47.01 ± 1.82^a^	1.65 ± 0.48^a^	1.31 ± 0.47^ab^	35.15 ± 0.14^a^	34.12 ± 0.10 ^ab^
*T*_3_	9.85 ± 0.15^a^	6.53 ± 0.24^ab^	47.41 ± 2.54^a^	44.93 ± 2.13^ab^	47.34 ± 2.54^ab^	44.94 ± 2.13^ab^	1.46 ± 0.50^a^	1.09 ± 0.29^ab^	34.89 ± 0.14^a^	34.13 ± 0.12^a^
*T*_4_	8.62 ± 0.27^a^	7.48 ± 0.19^ab^	34.75 ± 3.54^a^	30.10 ± 2.12^ab^	34.66 ± 3.54^a^	33.26 ± 2.12^a^	1.81 ± 0.39^a^	0.96 ± 0.17^ab^	34.91 ± 0.13^a^	35.30 ± 0.13^a^
*T*_5_	8.54 ± 0.17^a^	7.54 ± 0.23^ab^	34.91 ± 2.48^a^	28.89 ± 2.30^ab^	34.87 ± 2.48^a^	33.42 ± 2.30^a^	1.56 ± 0.50^a^	0.87 ± 0.33^ab^	34.90 ± 0.11^a^	35.31 ± 0.13^a^

Comparison with *T*_0_,^a^*P* < 0.05.; comparison with simultaneous point A group ^b^*P* < 0.05.

**Table 4:** The use of opioids between two groups of patients.

	Control	Treated	*t* value	*P* value
*T*_0_	21.87 ± 3.78	21.02 ± 3.81	1.112	0.536
*T*_3_	7.51 ± 2.63^a^	5.57 ± 1.06^ab^	2.559	0.023

^a^ *P* < 0.05 compared with *T*_0_; ^b^*P* < 0.05 compared with simultaneous point A group.

**Table 5:** Serum inflammatory indexes during hospitalization between two groups of patients.

Groups	Control		Treated	
	*T*_0_	*T*_3_	*T*_0_	*T*_3_
WBC (^*∗*^10^9^/L)	6.59 ± 1.93	7.02 ± 1.75	6.61 ± 1.88	6.23 ± 1.60
Neutrophil (^*∗*^10^9^/L)	4.56 ± 1.37	4.24 ± 1.41	4.43 ± 1.52	4,37 ± 1.33
Lymphocyte (^*∗*^10^9^/L)	1.616 ± 0.65	1.73 ± 0.71	1.71 ± 0.55	1.61 ± 0.80
CRP (mg/L)	1.057 ± 0.43	0.69 ± 0.12^a^	1.06 ± 0.43	0.68 ± 0.18^ab^
ESR (*μ*g/ml)	19.61 ± 10.08	11.14 ± 2.21^a^	19.60 ± 9.98	9.01 ± 2.02^ab^
CGRP (ng/L)	4.11 ± 0.68	3.67 ± 0.56^a^	3.52 ± 0.78	1.89 ± 0.51^ab^
IL6 (pg/ml)	487.69 ± 35.27	298.98 ± 24.96^a^	493.16 ± 29.13	133.62 ± 22.46^ab^

^a^ *P* < 0.05 compared with *T*_0_; ^b^*P* < 0.05 compared with simultaneous point A group.

**Table 6:** The two groups of postherpetic neuralgia incidence rate.

	Control	Treated	Total	*P* value
SHN	32	32	64	>0.05
VAS ≥ 4	8	2	10	<0.001
PHN(%)	25%	6.25%	15.62%	<0.05

^*∗*^ *P* < 0.05 compared with simultaneous point A group.

## Data Availability

Some or all data generated or used during the study are available from the corresponding author upon request.

## References

[1] Le P., Rothberg M. (2019). Herpes zoster infection. *BMJ*.

[2] Johnson R. W., Rice A. S. (2014). Clinical practice. Postherpetic neuralgia. *New England Journal of Medicine*.

[3] Aamir A., Girach A., Sarrigiannis P. G. (2020). Repetitive magnetic stimulation for the management of peripheral neuropathic pain: a systematic review. *Advances in Therapy*.

[4] Moreira-Marconi E., Cristina Moura-Fernandes M., Moura-Fernandes M. C. (2019). Evaluation of the temperature of posterior lower limbs skin during the whole body vibration measured by infrared thermography: cross-sectional study analysis using linear mixed effect model. *PLoS One*.

[5] Alexandre D., Prieto M., Beaumont F., Taiar R., Polidori G. (2017). Wearing lead aprons in surgical operating rooms: ergonomic injuries evidenced by infrared thermography. *Journal of Surgical Research*.

[6] Polidori G., Marreiro A., Pron H. (2016). Theoretical modeling of time-dependent skin temperature and heat losses during whole-body cryotherapy: a pilot study. *Medical Hypotheses*.

[7] Dworkin R. H., Gnann J. W., Oaklander A. L., Raja S. N., Schmader K. E., Whitley R. J. (2008). Diagnosis and assessment of pain associated with herpes zoster and postherpetic neuralgia. *The Journal of Pain*.

[8] Kawai K., Gebremeskel B. G., Acosta C. J. (2014). Systematic review of incidence and complications of herpes zoster: towards a global perspective. *BMJ Open*.

[9] Forbes H. J., Bhaskaran K., Thomas S. L. (2016). Quantification of risk factors for postherpetic neuralgia in herpes zoster patients: a cohort study. *Neurology*.

[10] Takao Y., Okuno Y., Mori Y., Asada H., Yamanishi K., Iso H. (2018). Associations of perceived mental stress, sense of purpose in life, and negative life events with the risk of incident herpes zoster and postherpetic neuralgia: the shez study. *American Journal of Epidemiology*.

[11] Finnerup N. B., Kuner R., Jensen T. S. (2020). Neuropathic Pain: From Mechanisms to Treatment.

[12] Forstenpointner J., Rice A. S. C., Finnerup N. B., Baron R. (2018). Up-date on clinical management of postherpetic neuralgia and mechanism-based treatment: new options in therapy. *The Journal of Infectious Diseases*.

[13] Schmader K. (2016). Herpes zoster. *Clinics in Geriatric Medicine*.

[14] Colloca L., Ludman T., Bouhassira D. (2017). Neuropathic pain. *Nature Reviews Disease Primers*.

[15] Hunter P., Fryhofer S. A., Szilagyi P. G. (2020). Vaccination of adults in general medical practice. *Mayo Clinic Proceedings*.

[16] Facchini G., Spinnato P., Guglielmi G., Albisinni U., Bazzocchi A. (2017). A comprehensive review of pulsed radiofrequency in the treatment of pain associated with different spinal conditions. *British Journal of Radiology*.

[17] Hermanns H., Hollmann M. W., Stevens M. F. (2019). Molecular mechanisms of action of systemic lidocaine in acute and chronic pain: a narrative review. *British Journal of Anaesthesia*.

[18] Moulin D. E., Morley-Forster P. K., Pirani Z., Rohfritsch C., Stitt L. (2019). Intravenous lidocaine in the management of chronic peripheral neuropathic pain: a randomized-controlled trial. *Canadian Journal of Anesthesia*.

[19] Uhl R. L., Roberts T. T., Papaliodis D. N., Mulligan M. T., Dubin A. H. (2014). Management of chronic musculoskeletal pain. *Journal of the American Academy of Orthopaedic Surgeons*.

[20] Rezaee L., Manaheji H., Haghparast A. (2019). Role of spinal glial cells in excitability of wide dynamic range neurons and the development of neuropathic pain with the L5 spinal nerve transection in the rats: behavioral and electrophysiological study. *Physiology & Behavior*.

[21] Cohen S. P., Mao J. (2014). Neuropathic pain: mechanisms and their clinical implications. *BMJ*.

[22] Krames E. S. (2015). The dorsal root ganglion in chronic pain and as a target for neuromodulation: a review. *Neuromodulation: Technology at the Neural Interface*.

[23] Liem L., van Dongen E., Huygen F. J., Staats P., Kramer J. (2016). The dorsal root ganglion as a therapeutic target for chronic pain. *Regional Anesthesia and Pain Medicine*.

[24] Vanneste T., Van Lantschoot A., Van Boxem K., Van Zundert J. (2017). Pulsed radiofrequency in chronic pain. *Current Opinion in Anaesthesiology*.

[25] Fei Y., Huang B., Deng J., Xu L., Yao M. (2021). Efficacy of dorsal root ganglion pulsed radiofrequency combined with paravertebral injection of recombinant human interferon-*α*2b in herpetic neuralgia. *Journal of Pain Research*.

[26] Huang J., Yang S., Yang J. (2020). Early treatment with temporary spinal cord stimulation effectively prevents development of postherpetic neuralgia. *Pain Physician*.

[27] Koga R., Yamada K., Ishikawa R., Kubota Y., Yamaguchi K., Iseki M. (2019). Association between treatment-related early changes in psychological factors and development of postherpetic neuralgia. *Journal of Anesthesia*.

[28] Ko E. J., No Y. A., Park K. Y., Li K., Seo S. J., Hong C. K. (2016). The clinical significance of infrared thermography for the prediction of postherpetic neuralgia in acute herpes zoster patients. *Skin Research and Technology*.

[29] Gross G. E., Eisert L., Doerr H. W. (2020). S2k guidelines for the diagnosis and treatment of herpes zoster and postherpetic neuralgia. *JDDG: Journal der Deutschen Dermatologischen Gesellschaft*.

[30] Hadley G. R., Gayle J. A., Ripoll J. (2016). Post-herpetic neuralgia: a review. *Current Pain and Headache Reports*.

[31] Zhu J., Fei Y., Deng J., Huang B., Yao M. (2020). Application and therapeutic effect of puncturing of the costal transverse process for pulsed radiofrequency treated T1-T3 herpes zoster neuralgia. *Journal of Pain Research*.

[32] Fujiwara A., Watanabe K., Hashizume K., Shinohara K., Kawaguchi M. (2018). Transforaminal vs. interlaminar epidural steroid injection for acute-phase shingles: a randomized, prospective trial. *Pain Physician*.

[33] O’Gara A., Leahy A., McCrory C., Das B. (2020). Dorsal root ganglion pulsed radiofrequency treatment for chronic cervical radicular pain: a retrospective review of outcomes in fifty-nine cases. *Irish Journal of Medical Science*.

[34] Ke M., Yinghui F., Yi J. (2013). Efficacy of pulsed radiofrequency in the treatment of thoracic postherpetic neuralgia from the angulus costae: a randomized, double-blinded, controlled trial. *Pain Physician*.

[35] Derruau S., Renard Y., Pron H. (2018). Combining magnetic resonance imaging (mri) and medical infrared thermography (mit) in the pre- and peri-operating management of severe hidradenitis suppurativa (Hs). *Photodiagnosis and Photodynamic Therapy*.

[36] Mosteller R. D. (1987). Simplified calculation of body-surface area. *New England Journal of Medicine*.

[37] Ding Y., Li H., Hong T., Yao P. (2020). Efficacy of pulsed radiofrequency to cervical nerve root for postherpetic neuralgia in upper extremity. *Frontiers in Neuroscience*.

[38] Wang M., Zhang J., Zheng L. (2021). Ultrasound-guided continuous thoracic paravertebral infusion of methylene blue in the treatment of postherpetic neuralgia: a prospective, randomized, controlled study. *Pain and Therapy*.

[39] Fei Y., Deng J., Lv H., Yao M., Wang T., Huang B. (2020). Pulsed radiofrequency of dorsal root ganglion of upper thoracic segment for herpes zoster neuralgia: case report. *Medicine (Baltimore)*.

[40] Vuka I., Marciuš T., Došenović S. (2020). Efficacy and safety of pulsed radiofrequency as a method of dorsal root ganglia stimulation in patients with neuropathic pain: a systematic review. *Pain Med*.

